# Evaluation of antibody and antigen cross-reaction in Kenyan dairy cattle naturally infected with two pestiviruses: Bovine viral diarrhea virus and classical swine fever virus

**DOI:** 10.14202/vetworld.2022.1290-1296

**Published:** 2022-05-24

**Authors:** Daniel Muasya, John Van Leeuwen, George Gitau, Shawn McKenna, Luke Heider, Joan Muraya

**Affiliations:** 1 Department of Health Management, Atlantic Veterinary College, University of Prince Edward Island (UPEI), Charlottetown, Prince Edward Island, Canada; 2 Department of Clinical Studies, Faculty of Veterinary Medicine, University of Nairobi, Nairobi, Kenya

**Keywords:** antibody, antigen, bovine viral diarrhea virus, classical swine fever virus, cross-reactivity, smallholder dairy

## Abstract

**Background and Aim:**

Bovine viral diarrhea virus (BVDV) and classical swine fever virus (CSFV) are important pathogens of cattle and pigs, respectively, and belong to the genus Pestivirus. As CSFV has been shown to infect cattle, it can create diagnostic challenges of BVDV results through possible cross-reactivity where cattle could be exposed to pigs and CSFV. This study aimed to determine the possible cross-reactivity of BVDV and CSFV enzyme-linked immunosorbent assay (ELISA) results for antigen (Ag) and antibody (Ab) among smallholder dairy cattle in Kenya.

**Materials and Methods:**

This was a cross-sectional study based on a single visit to farms to collect serum samples and other descriptive farm-level and animal-level information. Testing for BVDV Ag and Ab was conducted on serum samples from 320 dairy cows and heifers, with CSFV Ag and Ab testing conducted on a subset of 133 and 74 serum samples, respectively. CSFV testing was based on BVDV test results and the availability of enough sample volume from farms that kept pigs. The Ag and Ab tests utilized IDEXX ELISA for both BVDV and CSFV.

**Results:**

For the 74 samples with Ab tests for both viruses, 40 (54.0%) were BVDV Ab positive, while 63 (85.1%) were CSFV Ab positive. Of the 40 BVDV Ab positive samples, 36 cattle (90.0%) tested positive for CSFV Ab. However, of the 34 BVDV Ab negative samples, 27 (79.4%) were CSFV Ab test-positive. For the 133 samples with Ag tests for both viruses, 125 (94.0%) were BVDV Ag positive, while 2 (1.5%) samples were CSFV Ag positive. None of the eight BVDV Ag negative samples was positive for CSFV Ag and only two (1.6%) of the 125 BVDV Ag positive samples were positive for CSFV Ag.

**Conclusion:**

The results indicate either substantial cross-reactivity of the two Ab ELISA tests, or reactivity with some other protein in the samples that led to the positive Ab test results. There was only limited evidence for cross-reactivity of the two Ag ELISA tests. We recommend that Pestivirus genus cross-reactivity be considered when interpreting BVDV ELISA results in cattle, more for Ab than Ag tests. Further research is needed to clarify the levels of cross-reactivity between BVDV and other Pestivirus Ag and Ab tests from animals on mixed-species farms.

## Introduction

Bovine viral diarrhea virus (BVDV) is an economically important and genetically diverse member of the genus Pestivirus in the *Flaviviridae* family [1]. BVDV has two main strains, BVDV1 and BVDV2, with multiple sub-strains within them, and a third Hobi-like strain has been described recently [2,3]. There are two other Pestivirus species, classical swine fever virus (CSFV) and border disease virus (BDV) of importance to domestic animals [4]. Other species in the genus Pestivirus include pronghorn pestivirus, bungowannah virus, giraffe pestivirus, aydin-like pestivirus, rat pestivirus, and atypical porcine pestivirus (APP) [4-6]. The classification of Pestivirus genus was updated in the year 2017 and there is a new proposal to add eight new species recently described [7]. It has been demonstrated that the viruses in the Pestivirus genus have some level of genetic similarity with respect to diagnostic target molecules [8-10]. This similarity makes the molecular aspects of diagnosis and vaccine protectivity more difficult, with research focused on cross-protective vaccines and more specific diagnoses [11]. There is a very close similarity in the way Pestiviruses trigger immune-responses, thereby having the potential for cross-reaction on various tests, including enzyme-linked immunosorbent assays (ELISA) [12,13]. BVDV causes disease primarily in cattle; however, it has been shown to infect other mammalian hosts, such as pigs, camelids, and other domestic and wild ruminants across the globe [4]. The heterologous host infections may have analogous clinical and pathological syndromes to those in cattle [14]. Infections in multiple species could mean that some of these animals may be potential reservoirs or maintenance hosts, transmitting BVDV to cattle where there may be in contact, thereby hindering successful control [15,16].

Antibody (Ab) cross-reaction between CSFV and BVDV has been demonstrated as a challenge in diagnosing and monitoring BVDV in cattle herds [17,18]. Pestivirus Ab response for BVDV and CSFV infections in cattle has been shown to target glycoproteins E^rns^, E1, and E2, with the latter being more dominant [19,20]. The epitope mapping has been used to understand the cross-reactivity between the BVDV E2, CSFV E2, and BDV E2 glycoproteins as a means to identify monoclonal Ab domains [21,22]. These similarities pose a challenge to the serological diagnosis of both BVDV1, BVDV2, and CSFV [13,23]. The importance of BVDV in cattle populations is well documented, leading to direct and indirect losses to productivity and reproduction [24]. The disease has equally presented challenges with regard to a successful and sustainable control program [25]. Control programs have utilized diagnosis, vaccination, or both as key components, in addition to biosecurity [15]. For successful control, vaccination and serological diagnosis require more research on the genetic variation and cross-reactivity dynamics among pestiviruses [12,15], particularly in places that practice mixed livestock keeping of cattle, pigs, and small ruminants close contact, as is the often the case in Kenya. In a related primary research study preceding this work, there were 158 randomly selected farms in Meru County, Kenya. Among the 467 and 323 serum samples tested for BVDV antigen (Ag) and Ab using IDEXX ELISA tests, respectively. The seroprevalences of BVDV Ab and Ag were 47.1% (152/323) and 36.2% (169/467), respectively [26].

The previous reports of BVDV in other parts of Kenya have shown varying prevalence in cattle [26,27,28]; however, none has tested for the presence of cross-infection with CSFV between cattle and pig populations. In large-scale farms in the Rift Valley, Kenya, an Ab prevalence in dairy cows of 79.1% was recorded [27]. In Zebu cows in the Coastal area of Kenya, a 45.8% prevalence was recorded [29], but only 19.8% of Zebu cows were positive in western Kenya [28]. There is recent evidence of possible cattle infection with CSFV in China and India, which could further complicate the interpretation of the BVDV test results [30], if this means that the long-held belief that CSFV only infects swine is confirmed to be untrue.

This study aimed to determine the possible cross-reactivity of BVDV and CSFV ELISA results for Ag and Ab among dairy cattle.

## Materials and Methods

### Ethical approval

The research was approved on March 14^th^, 2019, by the University of Prince Edward Island (UPEI) Research Ethics Board (REB Ref # 6008082). Serum sampling in the primary study and laboratory testing in this study was carried out in accordance with UPEI animal use approval, and testing was done in accordance with standard laboratory operating procedures.

### Study period, area, and population

The study was conducted from May 2019 to April 2020 using unpublished data from a previous study [26]. The study location was in the Naari area of Meru County, Kenya. All the farms recruited were smallholder dairy farms and members of the Naari Dairy Farmers Cooperative Society. The cattle recruited were above 6 months of age, and the population consisted of mainly exotic breeds (with some local breeds) and their crosses. The farms practiced zero-grazing, communal grazing or a combination of both management practices.

### Experimental design

This was a cross-sectional study based on a single visit to the farm to collect serum samples and other descriptive farm-level and animal-level information. The sampling frame for the study was the 470 serum samples from the primary study conducted by Van Leeuwen *et al*. [26]. From that sampling frame, 320 samples that underwent testing for both BVDV Ag and Ab. Testing for CSFV Ag (n=133) and Ab (n=74) was conducted on a subset of the 320 samples, purposively selected, based on the following factors.

1. They came from animals on farms with at least one BVDV test positive sample in the primary study.
2. They came from farms rearing multiple animal species, especially pigs, in addition to cattle.
3. To identify CSFV exposure in cattle samples that were negative for BVDV Ab and Ag, a limited number of samples that were negative for BVDV Ab and Ag were tested.
4. Some samples were no longer available because they were used for other purposes.
5. Low volumes of sera remaining in the frozen sample vials made follow-up testing impossible for some samples.
6. Challenges with the freezers storing the frozen serum samples reduced the quality of the frozen sera for some samples.

**Figure-1:**
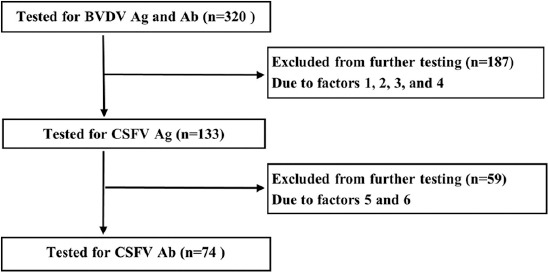
Flow diagram of samples used for testing for CSFV and BVDV Ag and Ab among purposively selected dairy cattle over 6 months of age in Kenya. CSFV=Classical swine fever virus, BVDV=Bovine viral diarrhea virus, Ag=Antigen, Ab=Antibody.

Figure-1 is a flow diagram of the sample numbers tested in relation to the above criteria.

### Laboratory testing

The serum samples were tested for antibodies and Ag for CSFV and BVDV using commercial ELISAs conducted at the Department of Clinical Studies, University of Nairobi. The University of Nairobi laboratory staff member was blinded to the identification of the tested animals and their other test results. The ELISA results produced an optical density, and then a sample-to-positive (S/P) ratio for each sample was calculated, indicating the reading that was adjusted for the positive control on the plate, after confirming that the negative control was in the correct range

The presence of BVDV Ag was tested with the BVDV Ag/Serum Plus Test^®^ kit (IDEXX Laboratories, Switzerland), according to the manufacturer’s instructions. Samples were considered positive when the S/P ratio was equal to or above 0.3. This test is reported to have a sensitivity of 98.7% and specificity of 95% and can detect the majority of BVDV 1 and 2 Ags.

For BVDV Ab testing, the BVDV Total Ab Test^®^ kit (IDEXX Laboratories) was used according to the manufacturer’s instructions, and an S/P ratio equal to or above 0.3 was considered positive. According to the manufacturer, this test is reported to have a sensitivity of 100% and specificity of 95% and can detect BVDV 1 and 2 antibodies. Samples with an S/P ratio of between 0.2 and 0.3 for both Ab and Ag were considered suspect according to the manufacture’s guidance.

CSFV Ag detection was conducted with CSFV Ag Serum Plus Test^®^ kits (IDEXX Laboratories) following the manufacturer’s instructions. Samples were considered positive when the S/P ratio was equal to or above 0.3. This test is reported to have a sensitivity of 90% and specificity of 100%. For CSFV Ab testing, CSFV Ab Test^®^ kits (IDEXX Laboratories) were used, according to the manufacturer’s instructions. Samples were considered positive when the S/P ratio was equal to or above 0.3. This kit is reported to have a sensitivity of 97.8% and specificity of 99.7%.

### Statistical analysis

Data were entered and organized into an Excel spreadsheet (Microsoft, Sacramento, California, USA). The CSFV data were merged with the BVDV data, by farm name and animal name. Descriptive statistics of cross-tabulations were carried out using STATA/IC 16.0 (StataCorp LLC, College Station, Texas, USA).

## Results

Of the 320 cattle that were tested for both BVDV Ab and Ag, 79 (24.3%) tested positive for BVDV Ab and Ag, and 87 (27.2%) tested negative for BVDV Ab and Ag (Table-1). There were 71 (22.2%) samples that were positive for BVDV Ab but negative for BVDV Ag, and 81 (25.3%) samples that were negative for BVDV Ab but positive for BVDV Ag. Three samples were suspect on the BVDV Ab test and were considered positive. Half (81) of the 162 BVDV Ag positive cattle were also positive for BVDV Ab, and 71 (45%) of the 158 BVDV Ag negative were BVDV Ab positive. Of the 133 CSFV Ag tests, only 2 (1.5%) samples were CSFV Ag positive (Table-2). None of the eight BVDV Ag negative samples were positive for CSFV Ag. Only two (1.6%) of the 125 BVDV Ag positive samples were positive for CSFV Ag.

**Table 1:** BVDV antibody and antigen ELISA results of 320 dairy cattle over 6 months of age from 134 randomly selected farms in Meru County, Kenya.

BVDV antigen	BVDV antibody			Total

	Negative	Positive	Suspect positive	
Negative	87	70	1	158
Positive	81	79	2	162
Total	168	149	3	320

BVDV=Bovine viral diarrhea virus, ELISA=Enzyme-linked immunosorbent assays

**Table 2:** CSFV antigen and BVDV antigen ELISA results of 133 purposively selected dairy cattle over 6 months of age in Meru County, Kenya.

CSFV antigen	BVDV antigen		Total

	Negative	Positive	
Negative	8	123	131
Positive	0	2	2
Total	8	125	133

CSFV=Classical swine fever virus, BVDV=Bovine viral diarrhea virus, ELISA=Enzyme-linked immunosorbent assays

Table-3 compares results from cattle that were tested for BVDV Ab and CSFV Ag, with 68 (51.9%) being BVDV Ab positive. The two cattle that were CSFV Ag positive were negative for BVDV Ab. None of the 68 BVDV Ab positive samples was positive for CSFV Ag. For the 74 CSFV Ab tests, 63 (85.1%) were CSFV Ab positive, and 40 (54.0%) were BVDV Ab positive (Table-4). Of the 40 BVDV Ab positive samples tested for CSFV Ab, 36 cattle (90.0%) tested positive for CSFV Ab. Of these 36 samples testing positive for BVDV and CSFV Ab, none tested positive for BVDV Ag or CSFV Ag.

**Table 3:** CSFV antigen and BVDV antibody ELISA results of 133 purposively selected dairy cattle over 6 months of age in Meru County, Kenya.

CSFV antigen	BVDV antigen		Total

	Negative	Positive	
Negative	63	68	131
Positive	2	0	2
Total	65	68	133

CSFV=Classical swine fever virus, BVDV=Bovine viral diarrhea virus, ELISA=Enzyme-linked immunosorbent assays

**Table 4:** CSFV antibody and BVDV antibody ELISA results of 74 purposively selected dairy cattle over 6 months of age in Meru County, Kenya.

CSFV antibody	BVDV antibody		Total

	Negative	Positive	
Negative	7	4	11
Positive	27	36	63
Total	34	40	74

CSFV=Classical swine fever virus, BVDV=Bovine viral diarrhea virus, ELISA=Enzyme-linked immunosorbent assays

Of the 34 BVDV Ab negative samples tested for CSFV Ab, 27 (79.4%) were CSFV Ab test positive (Table-4). Of the 63 CSFV Ab positive samples tested for BVDV Ab, 36 cattle (57.1%) tested positive for BVDV Ab. However, of the 11 CSFV Ab negative samples tested for BVDV Ab, 4 (36.4%) were BVDV Ab test positive. For the two samples that were positive for both CSFV Ag and BVDV Ag, an attempt was made to extract RNA from the samples to determine if they were truly CSFV or BVDV. Unfortunately, due to freezer storage issues, the sample quality was compromised, and therefore, it was not feasible to do RNA extraction.

## Discussion

This investigation of CSFV and BVDV Ab and Ag test results in dairy cattle in Kenya provides data on the frequency with which possible diagnostic cross-reactions may occur. As reported previously, the identification of pestiviruses by Ag or Ab ELISA has the potential for cross-reactivity due to the similarity in Pestivirus Ags and response antibodies used in various diagnostic tests [10,18,31]. There were 40 BVDV Ab positive cattle, with 36 of these (90.0%) cattle also testing positive for CSFV Ab (Table-4). The 36 samples testing positive for both BVDV and CSFV Ab, likely have been previously infected by either or both viruses. These results could suggest that there is substantial cross-reactivity of the two Ab ELISA tests, or the animals had antibodies to both CSFV and BVDV. Evidence of cross-reactivity between CSFV and BVDV complicating serological diagnosis has been demonstrated [21,32]. The antigenic epitope relied on for diagnosis is similar for BVDV and CSFV; thus, there is great potential for cross-reactivity [32].

It may also be possible that some other Pestivirus Ab in these samples leads to positive CSFV Ab test results and/or positive BVDV Ab test results. A study in Turkey reported that sheep and goats infected with BDV also tested positive for CSFV. Genetic sequencing of the DNA in the samples demonstrated the presence of Pestiviruses *Aydin/04* and *Burdur/05*, which were new variants of BDV [10]. There has been recorded evidence of cross-reactivity between other pestiviruses of importance to livestock, including BDV and APP, rendering diagnosis a challenge [33,34]. Cross-reactivity between BDV and BVDV has been reported to be a potential impediment in surveillance and diagnosis [35]. BDV has not been documented in Kenya, but it is thought to be present and may have impacted our study. Another Pestivirus genus member has been previously documented to be present in Kenya: Giraffe Pestivirus [26,36].

Of the 34 BVDV Ab negative samples, 27 (79.4%) were CSFV Ab positive. This result could mean that there were CSFV infections among the cattle population. CSFV infections have been demonstrated in cattle populations, as well as BVDV infections in pig populations, in other studies in different places globally [12,30,32,37,38]. It has been shown that BVDV infection in pigs is a challenge to the diagnosis of CSFV in pig herds where pigs and cattle are kept in close proximity [13,39]. This result means that cattle and pigs reared together can compound the maintenance of both pathogens and thus possible cross-reaction or co-infection

Only two (1.6%) of the 125 BVDV Ag positive samples were positive for CSFV Ag (Table-2). Therefore, it would seem that there is not much cross-reactivity between the two Ag ELISA tests. Other studies have reported CSFV Ags in cattle serum using Ag capture ELISA [37]. Given that cattle may become infected with CSFV, it is also possible that these two samples were from cattle that were infected with BVDV and CSFV at the time of sampling. A study of pestiviruses in Asia showed that there was about 30% genetic divergence between BVDV and CSFV heterogeneity, concluding that these two different Pestivirus species and genomic clusters were heterogeneous [17]. However, the genetic variation between BVDV 1 and 2, which are also classified as distinct species, has been shown to be quite homogenous, leading to some level of diagnostic cross-reactivity and immunologic cross-productivity [15,40]. Unfortunately, due to freezer issues, the sample quality was compromised; therefore, it was not feasible to do RNA extraction.

The high seroprevalences of BVDV Ab and Ag reported in the preceding primary study by Van Leeuwen *et al*. [26] approached or exceeded those of other reports [41,42]. Having a third of tested cattle testing positive for BVDV Ags [26] was surprising, suggesting that a substantial proportion of cattle had either transient or persistent infections of BVDV at the time of blood sampling, despite showing little or no clinical signs of BVDV disease. BVDV test results may be partly a function of test cross-reaction with CSFV since this has been demonstrated in other studies [13,21,22]. BVDV can easily be transmitted between cattle through body secretions and BVDV antibodies can remain in circulation for long periods of time [43-45]. A study looking at *Neospora caninum* and BVDV in export cattle from Rio Grande, Brazil found 75.36% being positive for BVDV Ags [46]. The prevalence of BVDV had been reported to be high in situations where mixed livestock are kept together and where wildlife and domestic cattle co-mingle [13,25,47]. Many smallholder dairy farms (SDF) in Kenya have these conditions; therefore, there is the possibility of co-circulation of BVDV among other pathogens on Kenyan SDFs [28,48]. In the primary study by Van Leeuwen *et al*. [26], pigs were identified to be an important associated exposure with the odds of samples testing positive for BVDV Ag being 6.1 times higher on farms with pigs than farms without pigs (p=0.02).

## Conclusion

Our findings demonstrate the challenges of interpreting Ab test results for BVDV and CSFV on farms where livestock species mingle on the same farm. The results indicate either substantial cross-reactivity of the two Ab ELISA tests, possible combined infections, or reactivity with some other Pestivirus in the samples, such as BDV, that led to positive CSFV Ab test results and/or positive BVDV Ab test results. There was only limited evidence for cross-reactivity of the two Ag ELISA tests.

This study was limited by using a subset of samples tested for CSFV that were originally tested for BVDV Ag and Ab. Having a larger proportion of the original samples tested would likely have provided clearer results, but logistical challenges precluded more samples from being tested. It is unlikely that the reasons for samples not being tested by CSFV are related to the cross-reactivity being investigated, so there is unlikely to be a selection bias in the results. Further research is needed to quantify the proportion of BVDV Ag false positives due to other pestviruses. We recommend a study comparing the serological test to other more specific tests, such as RT-PCR and/or sequencing or virus neutralization. It could be good to explore and utilize improved diagnostic ELISA kits for cattle populations in Kenya, which could lead to a more accurate establishment of seroprevalence for CSFV and BVDV infection.

## Authors’ Contributions

DM, JV, GG, SM, and LH: Study design, data analysis, and manuscript writing. JM: Provided the samples from her primary study and was involved in writing the manuscript. All authors read and approved the final manuscript.
